# Intracranial *Mycoplasma hominis* infection following emergent craniectomy

**DOI:** 10.1016/j.idcr.2021.e01175

**Published:** 2021-06-08

**Authors:** Zahra Qamar, Stavropoula Tjoumakaris, Matthew A. Pattengill, Maliha Ahmed, Bryan Hess

**Affiliations:** a Division of Infectious Diseases, Thomas Jefferson University Hospital, United States; b Department of Neurosurgery, Thomas Jefferson University Hospital, United States; c Department of Clinical Microbiology, Thomas Jefferson University Hospital, United States

**Keywords:** Mycoplasma hominis, Cranial infection, Neurosurgery

## Abstract

We present a case of a young healthy female who developed recurrent cranial wound infections after a traumatic injury, the etiologic organism finally identified as *Mycoplasma hominis,* an uncommon and difficult to isolate bacterium.

## Case

A 25-year old woman with no pertinent past medical history presented intoxicated after a mechanical fall down a flight of 15 stairs, and was found to have fractures of the left temporo-parietal bone, with extension into the sinuses and acute epidural hematoma with midline shift. She emergently underwent left decompressive hemicraniectomy with duroplasty. Her initial post-surgical course was uncomplicated and she was discharged home after 5 days.

She returned 1 month later with acute onset expressive aphasia. At that time, she denied any fevers, chills, or night sweats, however she reported clear fluid drainage from her operative site. MRI with and without contrast of the brain revealed a collection concerning for subdural empyema at the site of the recent craniectomy. She underwent a cranial wound washout and purulence was noted in the subdural plane. Intraoperative specimens were negative on Gram stain and grew low quantities (single or few colonies) of normal skin flora including *Staphylococcus capitis, Cutibacterium acnes* (formerly *Propionibacterium acnes*) and *Staphylococcus epidermidis*. Initially she was treated empirically with IV vancomycin, however she was transitioned to daptomycin after failure to achieve therapeutic vancomycin troughs. Her aphasia resolved, and following her clinical improvement she was discharged on post-operative day 4 with a plan to complete a 6-week course of daptomycin.

**Fig. 1:**
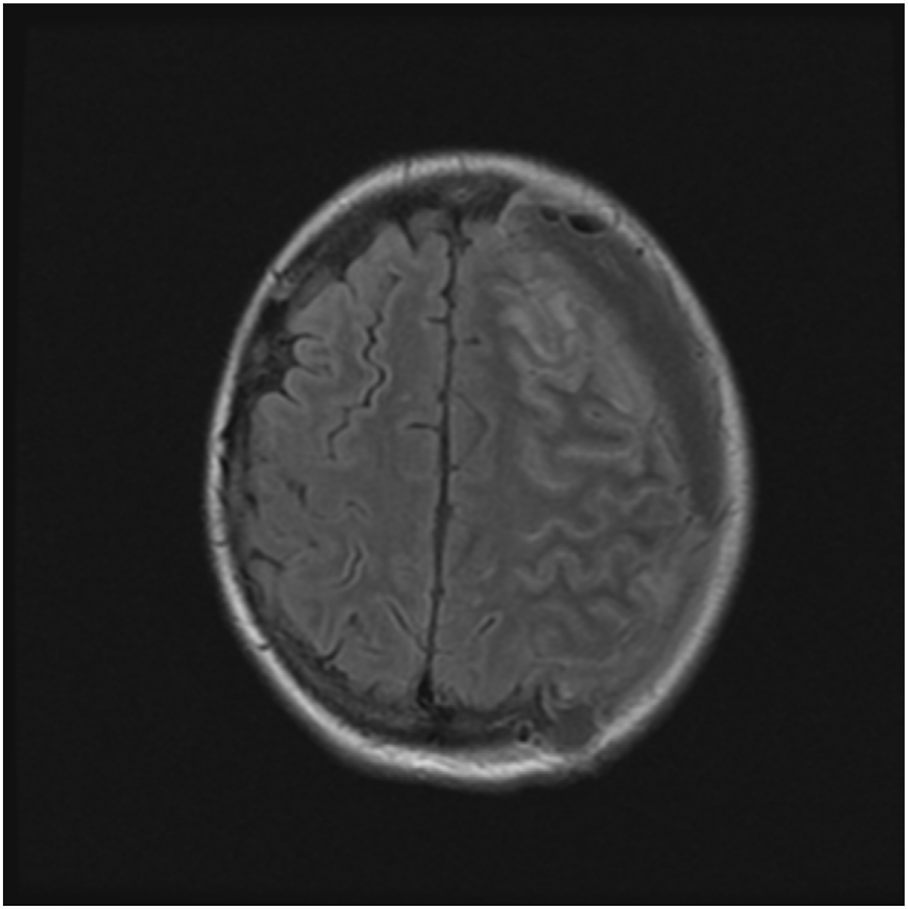
MRI Brain with and without contrast T2/FLAIR: Hyperintensity involving left frontal gyrus concerning for focal cerebritis of the left anterior frontal lobe.

Two days after her discharge, she was readmitted with fever, weakness, recurrent aphasia, and edema at her recent surgical site. MRI brain showed focal dehiscence along the temporal bone, leptomeningeal enhancement along the left cerebral hemisphere and a complex extra-axial fluid collection along the left cerebral convexity. There was no clinical or radiographic concern for osteomyelitis in several CT and MRI images enhanced with gadolinium. She was taken to the operating room urgently, and a large amount of purulent drainage was noted in the epidural, subdural, and subarachnoid spaces, without any adjacent bony involvement. These findings were consistent with a complex recurrent empyema and associated cerebritis. Daptomycin, cefepime and metronidazole were started empirically, however she failed to improve and developed worsening aphasia and right-hand weakness. Repeat MRI brain on post-operative day 4 demonstrated extensive leptomeningeal enhancement in the left cerebral hemisphere consistent with meningitis as well as swelling in the left frontoparietal region favoring cerebritis {Fig. 1}. Continuous EEG monitoring was consistent with subclinical seizures. Therefore, the patient was initiated on a short steroid taper and anticonvulsant medications.

**Fig. 2:**
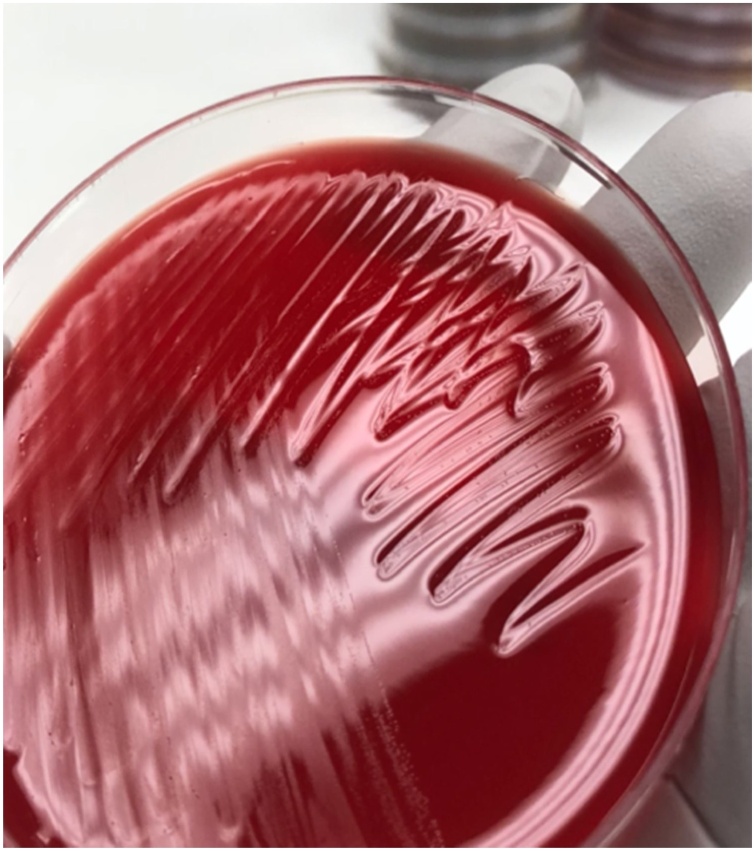
Pinpoint colonies visible on CDC anaerobic agar.

Gram stains from the intra operative specimens on this readmission revealed white blood cells (WBC) but no organisms and cultures showed no growth for the first 3 days. On day 4, possible pinpoint colonies were noted on anaerobic cultures (CDC anaerobic agar), however Gram staining of the possible colonies was negative. Colonies on CDC anaerobic agar from approximately day 6 of culture are shown in Fig. 2. Upon further review of cultures from the previous admission (collected 1 week prior), these pinpoint colonies were also identified. These findings of fastidious growth and inability to stain organisms with Gram stain raised suspicions for *Mycoplasma* or *Ureaplasma* infection and she was started on levofloxacin and doxycycline. Culture growth morphologically consistent with *Mycoplasma* species was recovered from all 9 intraoperative specimens (brain tissue, scalp, fluid aspiration) submitted for culture on readmission, and *Mycoplasma hominis* was eventually identified using 16 s ribosomal DNA sequencing. Susceptibility testing was requested, but unable to be obtained.

Her aphasia improved significantly 2 days after starting appropriate therapy. She was ultimately sent home to complete an 8-week course of levofloxacin 750 mg daily and doxycycline 100 mg every 12 h, and a 6 week course of daptomycin 10 mg/kg IV via PICC line. A follow-up MRI brain 1 month after completion of antibiotics revealed improvement in left cerebral edema and leptomeningeal enhancement. Her symptoms completely resolved and she completed her antibiotic regimen without complication. She did well, and 11 months later (delay due to COVID-19 pandemic) she underwent left autologous cranioplasty. She was seen 6 weeks post-operatively, and was doing well.

## Discussion

This case highlights an otherwise healthy young woman who presented on multiple occasions to the hospital with complications from a neurosurgery with an uncommon organism. Surgical site infections are not uncommon, and typical organisms are usually treated empirically until offending organisms are identified. Appropriate antimicrobial therapy also takes into account antibiotics penetration into abscesses in the cranial nervous system (CNS). Studies have suggested that the blood-brain barrier is different than the blood-CSF barrier and antimicrobial levels in the abscess cavities do not always correlate with the CSF levels [1]. Most common bacteria associated with post-operative cranial infections include *S.aureus*, followed by coagulase-negative staphylococci, *C.acnes*, other gram negative bacteria, while no organisms are identified in up to 8% of cases [2,3]. In the case of this patient, empiric therapies, followed by pathogen-directed antibiotics after the first infection resulted in recurrent hospitalization.

The diagnostic conundrum was caused by the unique characteristics of *Mycoplasma spp.* These organisms are distinctive among prokaryotes in that they do not have a cell wall (along with other *Mollicutes* genera such as *Ureaplasma*). Not only does this make them difficult to treat (most empiric antibiotic regimens target cell walls), it makes identification by Gram staining impossible [4].

The presence of colonies which could not be observed with Gram stain is what led the infectious disease and clinical microbiology team to suspect *Mycoplasma* species as a likely pathogen. Careful examination of microbiological cultures revealed pinpoint growth on culture media which could have been missed owing to their small size.

Prior to very recent phylogenetic updates [5] the genus *Mycoplasma* included 118 species, of which *M. genitalium* (now *Mycoplasmoides genitalium*), *M. hominis* (now *Metamycoplasma hominis*) and *M. pneumoniae* (now *Mycoplasmoides pneumoniae*) are clinically significant. These bacteria have trilayered cell membranes instead of a cell wall. They have the smallest genome of known self-replicating organisms in the bacterial kingdom, with limited biosynthetic capabilities which explains their parasitic or saprophytic existence and fastidious growth requirements which can complicate culture detection. Most mollicutes grow poorly or not at all on standard microbiologic media, however, *M. hominis* can grow on standard media (such as sheep’s blood trypticase soy agar) and may be detected as early as 2–5 days [4]. These bacteria can also be identified using polymerase chain reaction (PCR) and DNA sequencing when routine cultures identify no growth, or to confirm the identification of suspicious colonies as in our case.

Mycoplasmas are mucosally associated, rarely penetrating the submucosa, except in cases of immunosuppression or instrumentation when they can enter the bloodstream and disseminate. The natural habitat of *M. hominis* is the lower genital tracts of healthy sexually active adults and usually does not cause disease while *M. genitalium* is associated with non-gonococcal urethritis. *M. hominis* has been associated with bacterial vaginosis, pelvic inflammatory disease, and post-partum fever [6]. Infants can be colonized with genital mycoplasmas during delivery, and cases of neonatal meningitis, pneumonia and adverse pregnancy outcomes due to *M. hominis* have been reported due to vertical transmission [7,8].

Extrapulmonary and extragenital infections caused by mycoplasmas are infrequently encountered, and *M. hominis* is the most common culprit. There has been a recent rise in reported infections due to mycoplasma, likely as a result of more widespread use of PCR and DNA sequencing when routines cultures show no bacterial growth [4]. There are several case reports of cranial infections due to mycoplasmas. A healthy 29 year old male was found to have a brain abscess 3 weeks after craniotomy for a subdural hematoma due to a motor vehicle accident [9]. Brain empyema caused by *M. hominis* was identified in another 43 year old alcoholic male after sustaining a fall, when he developed signs of infection on day 5 of his admission [10]. Delayed intracranial surgical site infections are hypothesized to be disseminated possibly from iatrogenic genitourinary trauma, while infections presenting early on are potentially inoculated at the time of head trauma [11,12]. Meningitis as a delayed presentation after neurosurgical intervention for intracerebral hemorrhage has also been reported [13]. There are well documented cases of post-operative wound infections after open heart surgeries, causing sternal wound infections, mediastinitis and pericarditis. Other cases include sepsis syndromes, endocarditis and rarely abscesses [14]. These systemic infections are often linked to immunosuppression related to impaired cell-mediated immunity or hypogammaglobulinemia [15].

There have been no comparative studies to establish optimal therapeutic strategies for treatment of genital mycoplasmas. Standardized methods with defined MIC ranges have been published by the Clinical and Laboratory Standards Institute (CLSI) [16]. Mycoplasmas are intrinsically resistant to all beta-lactams, sulfonamides, trimethoprim and rifampin, with variable resistance of macrolides and lincosamides. *M. hominis* is intrinsically resistant to erythromycin and azithromycin owing to mutations in 23srRNA. Oral tetracyclines have been the drugs of choice for urogenital infections due to *M. hominis*, but resistance now occurs in 20–40 % of isolates and varies geographically. Fluoroquinolones are usually active against all mycoplasmas, however resistance in *M. hominis* has also been reported [[17], [18], [19]]. This has led to recommendations of obtaining in vitro susceptibility testing when *M. hominis* is recovered from normally sterile sites, from immunocompromised hosts, and/or from persons who have not responded to an initial treatment [17].

## Conclusion

This case illustrates the importance of considering *M. hominis* infection in all well-documented systemic and local infections in which properly collected specimens do not reveal a pathogen, while in the absence of prior antibiotic treatment. In this example, due to growth of skin flora that could be potentially pathogenic, an atypical infection was not initially suspected. On her second presentation, failure to grow bacteria despite copious purulence heightened the suspicion of an atypical infection and careful examination by an experienced pathologist ultimately led to the diagnosis. Review of literature also reveals a potential association of *M. hominis* infections with head trauma as illustrated in this case report, and has often caused delay in appropriate antimicrobials due to difficulty in identification of these bacteria.

## CRediT authorship contribution statement

**Zahra Qamar:** Writing - original draft, Writing - review & editing, Investigation. **Stavropoula Tjoumakaris:** Writing - review & editing. **Matthew A. Pattengill:** Writing - review & editing. **Maliha Ahmed:** Writing - original draft, Writing - review & editing. **Bryan Hess:** Writing - review & editing, Conceptualization.

## Declaration of Competing Interest

The authors report no declarations of interest.
